# Transducer of ERBB2.1 (TOB1) as a Tumor Suppressor: A Mechanistic Perspective

**DOI:** 10.3390/ijms161226203

**Published:** 2015-12-15

**Authors:** Hun Seok Lee, Juthika Kundu, Ryong Nam Kim, Young Kee Shin

**Affiliations:** 1 Research Institute of Pharmaceutical Science, Department of Pharmacy, College of Pharmacy, Seoul National University, Seoul 08826, Korea; ryanlee0@snu.ac.kr (H.S.L.); juthika23@snu.ac.kr (J.K.); ryongnamkim@gmail.com (R.N.K.); 2 Tumor Microenvironment Global Core Research Center, Seoul National University, Seoul 08826, Korea; 3 The Center for Anti-cancer Companion Diagnostics, School of Biological Science, Institutes of Entrepreneurial BioConvergence, Seoul National University, Seoul 08826, Korea

**Keywords:** anti-proliferative, apoptosis, invasion, migration, transducer of erbb2.1, tumor suppressor

## Abstract

Transducer of ERBB2.1 (TOB1) is a tumor-suppressor protein, which functions as a negative regulator of the receptor tyrosine-kinase ERBB2. As most of the other tumor suppressor proteins, TOB1 is inactivated in many human cancers. Homozygous deletion of *TOB1* in mice is reported to be responsible for cancer development in the lung, liver, and lymph node, whereas the ectopic overexpression of TOB1 shows anti-proliferation, and a decrease in the migration and invasion abilities on cancer cells. Biochemical studies revealed that the anti-proliferative activity of TOB1 involves mRNA deadenylation and is associated with the reduction of both cyclin D1 and cyclin-dependent kinase (CDK) expressions and the induction of CDK inhibitors. Moreover, TOB1 interacts with an oncogenic signaling mediator, β-catenin, and inhibits β-catenin-regulated gene transcription. TOB1 antagonizes the v-akt murine thymoma viral oncogene (AKT) signaling and induces cancer cell apoptosis by activating BCL2-associated X (BAX) protein and inhibiting the BCL-2 and BCL-XL expressions. The tumor-specific overexpression of TOB1 results in the activation of other tumor suppressor proteins, such as mothers against decapentaplegic homolog 4 (SMAD4) and phosphatase and tensin homolog-10 (PTEN), and blocks tumor progression. TOB1-overexpressing cancer cells have limited potential of growing as xenograft tumors in nude mice upon subcutaneous implantation. This review addresses the molecular basis of TOB1 tumor suppressor function with special emphasis on its regulation of intracellular signaling pathways.

## 1. Introduction

**Figure 1:**
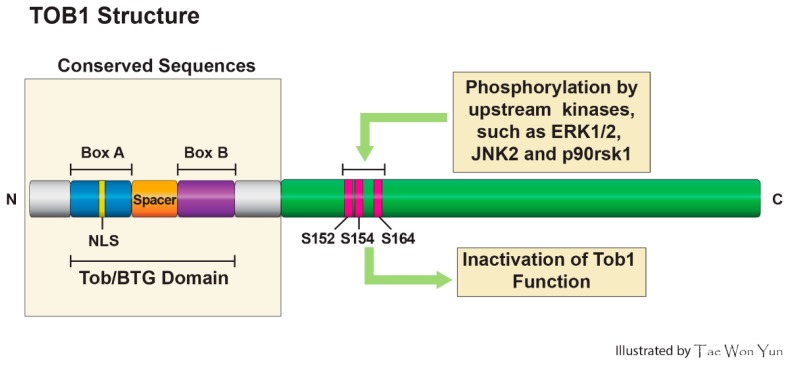
Structural features of TOB1 protein and its phosphorylation sites.

The protein encoded by the transducer of ERBB2.1 (*TOB1*) is a member of the TOB/B cell translocation gene (BTG) family, which also consists of *BTG1*, *BTG2*/*TIS21*/*PC3*, *BTG3*, *BTG4*/*PC3B*, and *TOB2* [1]. These family proteins are characterized by a N-terminal TOB/BTG homology domain which contains two short elements, Box-A (harboring Nuclear localization sequence (NLS)) and Box-B, which are a highly conserved and a non-conserved spacer sequence inserted between the two conserved A and B boxes [2] (Figure 1). According to a recent report, the conserved N-terminal domain is a module for protein-protein interaction involved in binding to the paralogues CNOT7 (human Caf1/Caf1a) and CNOT8 (human Pop2/Calif/Caf1b), the deadenylase components of the CCR4-NOT complex, whereas the C-terminal region is less conserved and mediates unique protein-protein interactions specific to each family member. Structural analysis of the BTG domain implies the existence of a high degree of similarity between TOB1 and TOB2, and between BTG1 and BTG2, whereas BTG3 and BTG4 are less related to its members [3]. Among TOB/BTG protein family members, TOB1 and TOB2 have the longest C-terminal domains, involved in protein-protein interactions, which plays a pivotal role for transcription, mRNA turnover, and other regulatory functions. Because of their well-defined role in the inhibition of cell proliferation, TOB/BTG proteins are also known as anti-proliferative (APRO) family proteins [4]. In 1991, Bradbury *et al.* reported the first member of the TOB/BTG family, which was isolated from rat pheochromocytoma cells and named PC3 (pheochromocytoma cell-3) [5]. In the same year, Fletcher *et al.* reported a mouse *Pc3* homolog from 12-*O*-tetradecanoyl phorbol-13-acetate (TPA)-stimulated mouse NIH3T3 cells and named it TPA-induced sequence 21 (*Tis21*) [6]. In the following year, a successful molecular cloning of B cell translocation gene-1 (*BTG1*) from B cell lymphocytic leukemia, presenting 65% homology with *Tis21*/*PC3*, was reported [7]. Later on, Rouault *et al.* cloned *BTG2* as the human homolog of the *PC3*/*Tis21* gene [8]. In the same year, Matsuda and colleagues reported the cloning of a cDNA encoding a novel protein termed TOB1, which exhibited sequence homology to known anti-proliferative gene product BTG-1 in its nearly half N-terminal. Sequencing analysis of the nucleotide revealed that TOB1 is a 45 kDa protein that lacks a Src Homology 2 (SH2) or SH3 domain. While the N-terminal half of TOB1 showed sequence similarity with BTG1, the C-terminal half is elucidated to have a unique proline and glutamine [9]. This family has about 20 members which have been discovered from different species, ranging from nematodes to human, all retaining phylogenetically conserved homology [10]. The *TOB1* transcripts are expressed in adult brain tissues, in which they could play pivotal roles in learning and memory [11]. Similar to the *TOB1*, *BTG2* mRNAs are elevated in the ventricular zone of the central nervous system (CNS), including the brain [12]. Additionally, BTG2 inhibits medulloblastoma, a very aggressive tumor of the cerebellum, by inhibiting proliferation and triggering the differentiation of the precursors of cerebellar granule neurons [13]. The *TOB1* gene is mapped on chromosome 17q, which harbors a series of tumor suppressor genes. The inactivation of tumor suppressor genes located on chromosome 17 is associated with stomach and lung cancer. In a pioneering study, Yoshida and colleagues [14] first reported that *TOB* gene knockout mice show spontaneous tumor formations primarily in the lung, liver, and lymph nodes. Others reported that TOB1 restoration inhibits growth factor receptor ERBB2-mediated signaling [9] and attenuates tumor cell survival [15]. Accumulating evidence suggests that TOB1 functions as a tumor suppressor protein. The purpose of this review is to delineate the molecular mechanisms underlying TOB1 tumor suppressor functions.

## 2. Status of TOB1 Expression in Human Cancers

Like many other tumor suppressor proteins, down-regulated TOB1 expression was reported in various cancers, mostly in breast [16], pancreas [17], thyroid [18], and stomach [19]. The loss of heterozygosity (LOH) of *TOB1* mapped on chromosome 17q is also common in human lung cancer tissue. Immunohistochemical analysis of human lung cancer tissues revealed decreased expression of TOB1 in 72% of patients with squamous cell carcinoma. According to this study, elevated expression of phosphorylated TOB1 (at serine 152 and serine 154 residues), an inactive form of the protein, was only observed in lung cancer tissues compared to adjacent normal tissues [14]. The increased incidence of gastric cancer is also correlated with the inactivation of tumor suppressor genes, including *TOB1* on chromosome 17. The LOH of *TOB1* was also detected in human gastric cancer specimens. This study reported that TOB1 expression was either abolished or reduced in 75% of patients with gastric cancer [19]. The phosphorylation-mediated inactivation of TOB1 has been reported. Yu *et al.* [19] reported that the ratio of unphosphorylated and phosphorylated TOB1 is reduced in differentiated gastric cancer cells, suggesting that the portion of TOB1 inactivation is increased in gastric cancer [19]. The phosphorylation-mediated inactivation of TOB1 is more frequent in papillary thyroid carcinoma than that in follicular thyroid carcinoma. The levels of TOB1 phosphorylation in papillary thyroid cancer are directly associated with tumor size, metastasis, and the presence of poorly differentiated lesions. In anaplastic thyroid carcinomas, TOB1 has been largely detected in the non-phosphorylated state, but with a notable reduction in protein expression [18].

The inactivation of TOB1 is also observed in breast carcinogenesis. LOH on chromosome 17p has been reported in more than 40% of sporadic breast carcinomas, implying the presence of putative tumor suppressor genes on this chromosome arm [20]. Microarray analysis of 25 human tumors with primary breast cancers and subsequent biochemical analysis revealed that, in most of the breast cancers and lymph nodes, expression levels of *TOB1* mRNA and protein are altered [21]. Furthermore, TOB1 expression levels are inversely associated with the tumorigenicity and metastatic ability of breast cancer cell lines as well as with tumor progression in patients with breast cancers [21]. All these results highly suggest that the loss of TOB1 expression plays a vital role in breast cancer progression.

## 3. Underlying Mechanisms of TOB1 Inactivation

**Figure 2:**
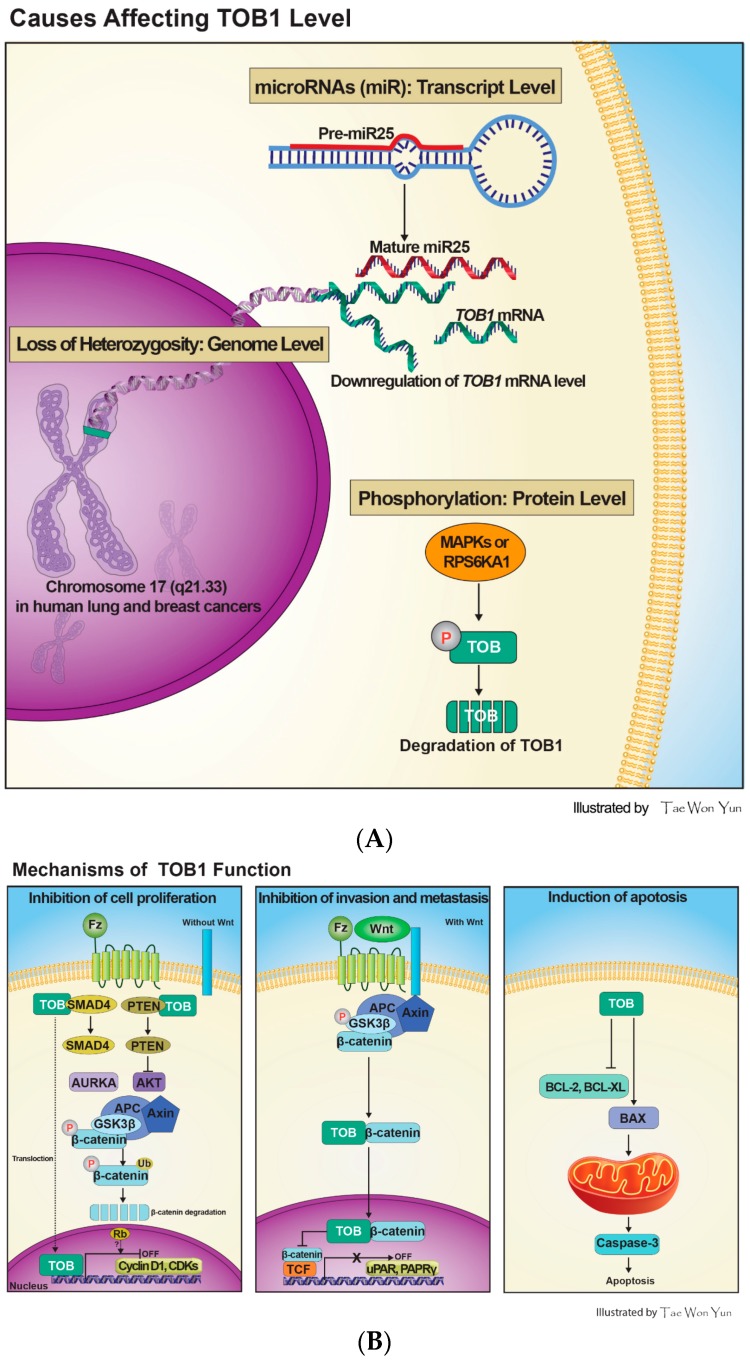
Processes affecting TOB1 levels and diverse mechanisms underlying TOB1 tumor suppression activity. (**A**) The level of TOB1 expression is affected by three different mechanisms, including LOH for *TOB1* gene in the cancer genome, down-regulation of *TOB1* mRNA expression by target microRNAs, and induction of TOB1 protein degradation by cues such as its phosphorylation. *miR25* represses *TOB1* mRNA expression by binding to its 3′-untranslated region (3′-UTR) directly. TOB1 phosphorylated by MAPKs or RPS6KA undergoes ubiquitination by the ubiquitin ligase, SKP2, resulting in its degradation through the proteasomal pathway; (**B**) TOB1, a tumor suppressor protein, inhibits cancer cell proliferation, invasion, and metastasis and leads to the induction of apoptosis. In addition to inhibiting the transcription of cell cycle-associated genes in the nucleus, TOB1 physically cooperates with tumor suppressors, SMAD4 or phosphatase, and tensin homolog-10 (PTEN), to produce a yet unknown signal for the degradation of AURKA or AKT, thereby indirectly regulating β-catenin level in the cytosol. Moreover, TOB1 directly interacts with β-catenin in the nucleus, leading to down-regulation of target genes of the TCF/β-catenin complex. TOB1 overexpression induces the reduction of BCL-2 and BCL-XL expression, increment of BAX expression, and caspase-3 activation.

To date, several studies demonstrated the mechanisms by which TOB1 is inactivated (Figure 2A). In an effort to delineate the mechanisms by which ERBB2-associated TOB1 becomes inactivated, Suzuki *et al.* first demonstrated that TOB1 is phosphorylated on serine and threonine residues, but not on its tyrosine residue, by a ribosomal protein S6 kinase polypeptide 1 (RPS6KA1) [22]. A subsequent study revealed that TOB1 is inactivated during oncogenic *Ras*-induced cell transformation or growth factor stimulation through the phosphorylation of its serine-152, -154, and -164 residues by upstream kinases, extracellular signal-regulated kinase-1/2 (ERK1/2) [23]. Maekawa *et al.* [24] further demonstrated that TOB1 is a substrate of mitogen-activated protein kinase (MAPK) such as ERK1/2 and c-Jun-N-terminal kinase (JNK). According to this study, an ERK-binding or docking region is located in the TOB1 N-terminal domain, whereas the ERK-mediated phosphorylation of TOB1 occurs in its C-terminal domain. Upon phosphorylation, the quick cellular turnover of TOB1 is mediated by SKP2, a substrate-targeting subunit of the SCF (Skp1/Cul1/F-box protein) ubiquitin ligase complex as evidenced by the stabilization of the protein in both SKP2-null mouse fibroblasts and human cervical cancer (HeLa) cells lacking SKP2. SKP2 mutant, lacking the F-box or leucine-rich repeat, was unable to interact with TOB1, and cells transfected with the SKP2 mutant failed to induce TOB1 ubiquitination [25]. It has been reported that the proteasomal degradation of TOB1 occurs upon polyubiquitination of the protein. However, a recent study by Suzuki *et al.* [26] demonstrated that, even in absence of E3 ligases, TOB family proteins may undergo monoubiquitination on two lysine residues (Lys48 and Lys63) located in the TOB/BTG homology region. A TOB1 mutant (both Lys48 and Lys63 are substituted with alanine) undergoes stronger polyubiquitination than wild-type TOB1 *in vivo*, suggesting that TOB1 monoubiquitination can prevent its further ubiquitination, thereby causing the stabilization of the protein.

There has been emerging evidence of the involvement of certain micro-RNAs (miR) in the down-regulation of *TOB1* expression. A recent study reported that miR-25, which is highly expressed in the plasma and primary tumor tissues from patients with gastric cancer, repressed *TOB1* mRNA expression through direct interaction with its 3′-untranslated region (3′-UTR), thereby increasing the proliferation, metastasis, and invasion of gastric cancer cells [27].

## 4. Mechanisms of TOB1 Tumor Suppressor Functions

TOB1 tumor suppressor function has been largely confirmed by its anti-proliferative and apoptosis-inducing effects on various cancer cells. Recent studies demonstrated that loss of TOB1 facilitates migration and invasion of human lung cancer [28] and gastric cancer [29] cells, indicating the role of TOB1 in suppressing tumor progression. The following section will shed light on the molecular mechanisms associated with TOB1 tumor suppressor function (Figure 2B).

### 4.1. Role of TOB1 in Cell Cycle Regulation

Cellular quiescence, a dormant state of the cell cycle, is characterized by reduced cell size and metabolic activity. TOB1 plays a pivotal role in maintaining cells in a quiescent state, thereby inhibiting cellular proliferation [4]. The suppression of T cell activation and cytokine production by ectopic expression of TOB1 is associated with reduced transcriptional activation of interleukin (*IL*)*-2* [30]. According to this study, TOB1 interacted with another tumor suppressor protein, SMAD4, and enhanced the binding of SMAD4 to the SMAD binding element (SBE) located on the promoter region of *IL-2*, thereby dampening *IL-2* transcription. We also reported that TOB1 overexpression in human gastric cancer (MKN28 and AGS) cells restored the levels of SMAD4 and increased the promoter activity and the mRNA and protein level of its target gene *CDKN2B* (*P15(INK4B*)), thereby inducing cell cycle arrest and apoptosis [29]. The role of TOB1 in SMAD4 regulation was further confirmed by silencing TOB1 in MKN1 and AGS cells, which showed diminished expression of SMAD4 and CDKN2B. Thus, TOB1 may exert its anti-proliferative effects by activating the SMAD4-mediated signaling [29].

The anti-proliferative properties of TOB1 are regulated by various signaling pathways affecting cell cycle progression. Yosida *et al.* [31] demonstrated that homozygous deletion of *TOB1* in mice increased the expression and the promoter activity of CCND1 (cyclin D1) via interaction with HDAC1 (histone deacetylase 1). Ectopic expression of TOB1 reduced the expression and promoter activity of CCND1 in human embryonic kidney (HEK293) cells [31]. Hiramatsu *et al.* [25] also reported that TOB1 stabilization in HeLa cells lacking SKP2, a protein responsible for TOB1 degradation, or in SKP2-null mouse fibroblasts was positively correlated with reduced CCND1 expression and cell proliferation. Likewise, CCND1 expression was elevated in serum-starved *TOB1*-null mouse embryonic fibroblasts and the re-introduction of either wild-type TOB1 or TOB1 mutants on phosphorylation-responsive serine residues with alanine restored its ability to suppress CCND1 expression [23]. Helms *et al.* suggested that TOB1 phosphorylation prevents the anti-proliferative role of TOB1 in HER2 and/or EGFR-positive breast cancers, implying that phosphorylated TOB1 is an inactive form [32]. However, TOB1 failed to inhibit the growth of cells with deficient RB (retinoblastoma protein), suggesting that TOB1 functions upstream of RB in regulating the cell cycle [23]. Similarly, a RB-dependent G1/S phase cell cycle arrest was mediated by BTG2, another member of the PC3/BTG2/TOB family proteins. Removal of residues 50 to 68 from the BTG2 abrogated its anti-proliferative activity [33]. Since residues 50 to 68 constitute a conserved domain in the PC3/BTG/TOB gene family, the anti-proliferative effect of TOB1 may be linked to this conserved domain. The transcriptional activation of CCND1 is regulated by TCF (T-cell factor)/CTNNB (β-catenin). TOB1 overexpression in HeLa cells showed increased interaction between TOB1 and CTNNB and, subsequently, decreased the transcriptional activity of CTNNB [34]. Since TOB1 competes with LEF/TCF factors for binding with CTNNB, the formation of the TOB1-CTNNB complex may block the formation of the TCF/CTNNB complex and may lead to the inhibition of CCND1 expression. We reported that TOB1 overexpression reduced the viability of MKN28 and AGS cells by blocking CTNNB-mediated signaling. According to our previous study, TOB1 overexpression in MKN28 and AGS cells inhibited CTNNB protein expression, decreased the *CTNNB* reporter gene activity, and reduced CCND1 expression via blockade of AKT phosphorylation [29]. Moreover, silencing *TOB1* in MNK1 and AGS cells restored CTNNB levels, thereby increasing CCND1 expression [29].

**Figure 3:**
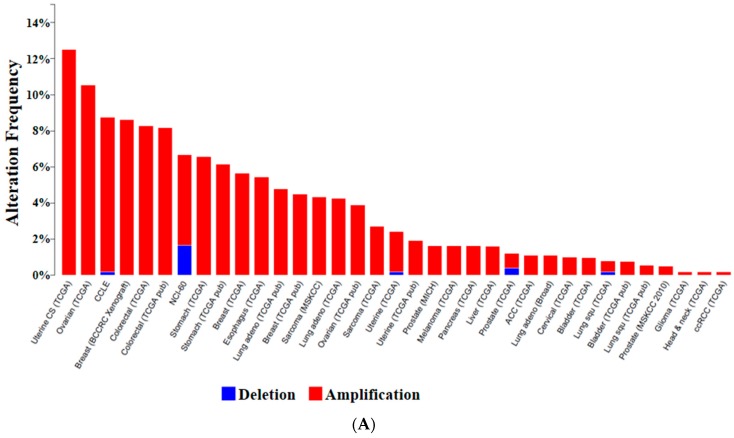

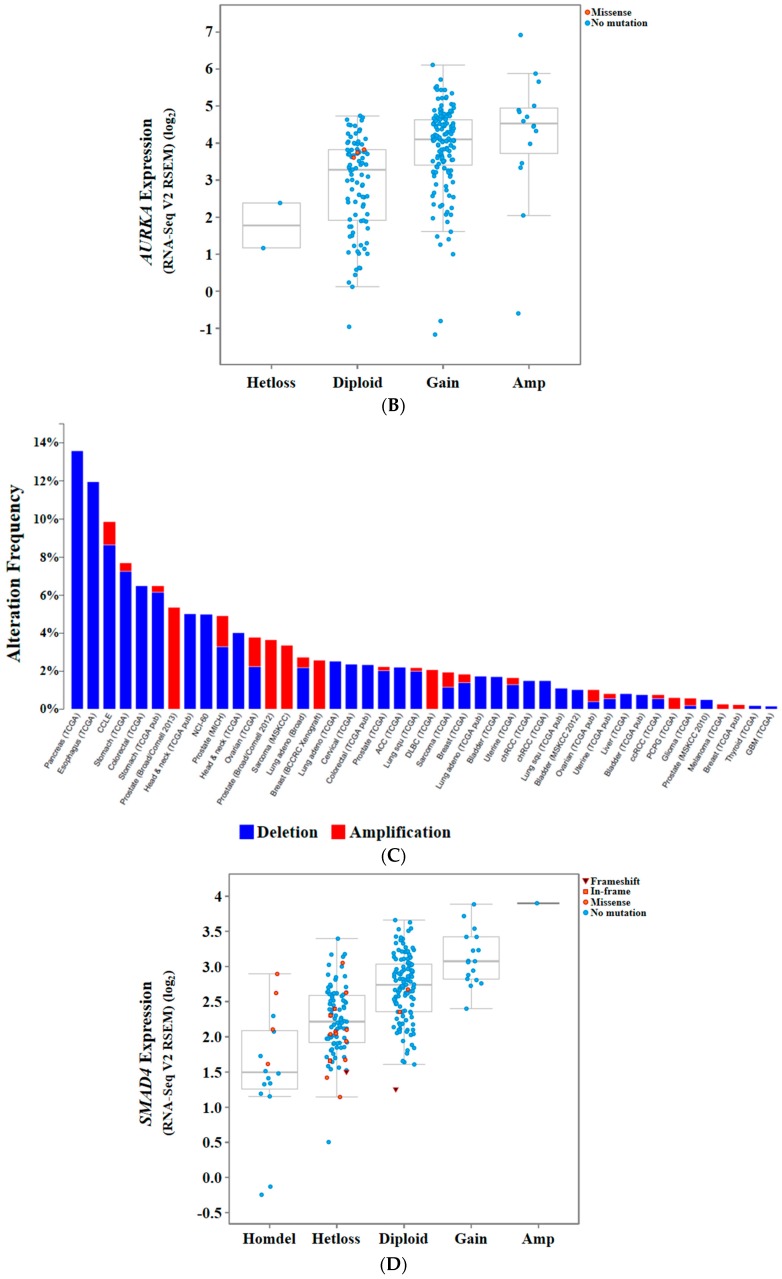

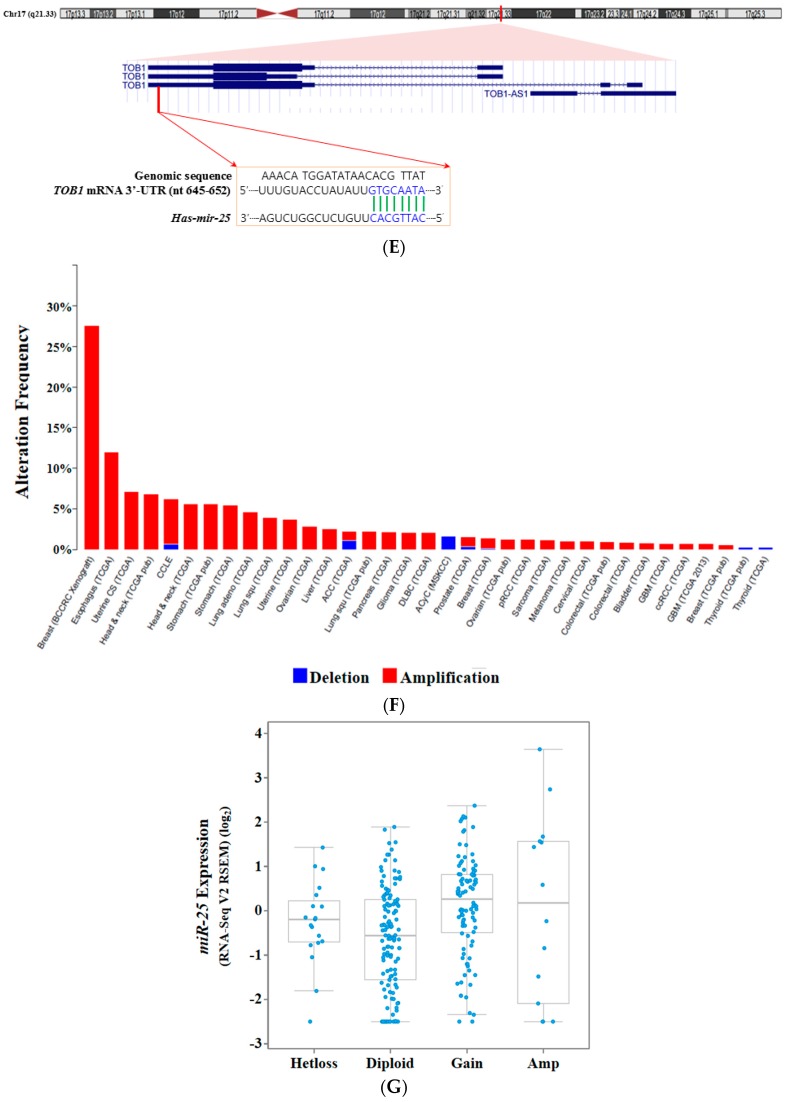
Genomic copy number alteration and expression of *AURKA*, *SAMD4*, and *miR-25* in TCGA human clinical cancer samples across a variety of cancer types. (**A**) Genomic copy number amplification of *AURKA* in human clinical cancer samples; (**B**) Up-regulation of *AURKA* expression in human stomach cancer samples, showing its gain and amplification; (**C**) Genomic copy number deletion of *SAMD4* in human clinical cancer samples; (**D**) Down-regulation of *SAMD4* expression in human stomach cancer samples, showing its deletion; (**E**) Binding of *miR-25* to *TOB1* mRNA 3′-UTR; (**F**) Genomic copy number amplification of *miR-25* in human clinical cancer samples; and (**G**) Up-regulation of *miR-25* expression in human stomach cancer samples, showing its gain and amplification.

Another exciting novel discovery of a new signaling axis bridging the oncogenic CTNNB signaling pathway and TOB1 has been reported by our research group [28]. Though it was known that SMAD4 could suppress the oncogenic CTNNB signaling pathway, the detailed molecular mechanism of how SMAD4 could exert its suppressive role on CTNNB signaling remained unclear until our report. We discovered that SMAD4 directly interacts with AURKA, thereby inducing AURKA degradation via the proteasomal pathway and consequently destabilizing CTNNB by removing GSK-3β phosphorylation by AURKA in dysplastic cells [28]. Consistent with the newly discovered oncogenic role of AURKA in the signaling axis connecting TOB1-SMAD4 with the CTNNB signaling pathway, we determined that the *AURKA* gene was highly amplified in human clinical cancer samples across a variety of cancer types in TCGA (The Cancer Genome Atlas) database (Figure 3A). We also demonstrated that *AURKA* mRNA expression was highly up-regulated in TCGA stomach cancer samples, showing its gain and amplification at the genomic level (Figure 3B). In addition, consistent with the tumor-suppressive role of SMAD4, the *SMAD4* gene locus showed homozygous deletions in those human clinical TCGA cancer samples and its mRNA expression was highly down-regulated in TCGA stomach cancer samples, indicating its deletion (Figure 3C,D). Our analysis of copy number alteration (CNA) in the *TOB1* genomic locus using these TCGA human clinical cancer samples did not reveal any significant characteristics, which is very consistent with the previous reports that *TOB1* mRNA could be degraded by miR-25 and that the TOB1 protein level is regulated by phosphorylation rather than by genomic copy number alteration [25,27]. Interestingly, we found that the *miR-25* locus was highly amplified in these TCGA human clinical cancer samples and *miR-25* expression was highly up-regulated in TCGA stomach cancer samples, showing its gain and amplification at the genomic level (Figure 3E–G). This result strongly suggests that the strong up-regulation of *miR-25* expression might be responsible for the down-regulation of TOB1 expression in cancer cells.

TOB1 also attenuated the expression of cyclin-dependent kinases (CDKs), thereby inhibiting cell proliferation. *TOB1* deletion increases hepatocyte proliferation by directly associating with ribonuclease protein CAF1 (carbon catabolite repressor (CCR)-associated factor-1) and CDK1, thereby modulating CDK1 kinase activity [35]. Although this finding has been implicated in TOB1-mediated liver regeneration, a similar phenomenon may promote proliferation of hepatocellular carcinoma cells. The 3′-poly(A) tail plays a vital role in the regulation of both the stability and the translational efficiency of eukaryotic mRNA [36,37]. TOB1 interaction with CAF1 deadenylase induces the destabilization of certain mRNAs encoding cell proliferation proteins and, hence, can inhibit cell proliferation [38]. For instance, CPEB (cytoplasmic polyadenylation element-binding protein) binds to *cis*-elements (CPEs) in the 3′-UTR of *c-MYC* mRNA, further recruiting CAF1 and TOB1, and forms the CPEB-TOB1-CAF1 complex, which could accelerate deadenylation and decay of *c-MYC* mRNA encoding the oncoprotein implicated in cell proliferation [39]. Another mechanism of TOB1 anti-proliferative effect is the induction of CDK inhibitors. Stable transfection of human breast cancer MCF-7 cells with TOB1 induced the expression of a CDK inhibitor CDKN1B (P27 (KIP1)), thereby attenuating the proliferation and growth of these cells [16]. EZH2 (enhancer of Zeste 2) knockdown, a growth stimulator, in colon cancer cells induces G1/S phase arrest in the cell cycle, concomitantly causing the elevated expression of *TOB1* [40]. A recent study indicated that TOB1 may be involved in the inhibition of human embryonic cancer stem cell proliferation as the depletion of Nanog, a key transcription factor that maintains pluripotency, increased TOB1 expression and down-regulated the expression of cyclins, CDK1 and CDK6 [41].

The anti-proliferative activity of TOB1 is attributed to the nucleo-cytoplasmic translocation of the protein. TOB1 contains nuclear localization signal (NLS, residues 18–40) and nuclear export signal (NES, residues 2–14) domains that regulate the nuclear-cytoplasmic trafficking of the protein. To examine the correlation between the anti-proliferative activity and subcellular localization of TOB1, Maekawa *et al.* [42] exogenously added the NLS or NES signal sequences to TOB1 and demonstrated that the ability of exogenously added NLS-TOB1 to inhibit cell cycle progression from G0/G1 to S phase was weakened, suggesting that TOB1 cytoplasmic retention is associated with its anti-proliferative role. In contrast, TOB1 presenting mutations in the NLS sequence is retained in the cytoplasm, which results in the impairment of its anti-proliferative activity [43]. Moreover, the blockade of nuclear export in cells transfected with TOB1-NES mutant inhibited cell proliferation. According to this study, TOB1 anti-proliferative activity is dependent on its nuclear localization [43]. The discrepancy in TOB1 subcellular distribution and its impact on cell proliferation is still unclear and warrants further study.

### 4.2. Role of TOB1 in the Induction of Apoptosis

Besides the inhibition of cell proliferation, TOB1 also induces apoptosis. TOB1 overexpression in MCF-7 cells attenuated the expression of anti-apoptotic protein, BCL2 and BCL-XL, whereas *TOB1* knockdown by short-hairpin RNA (shRNA) increased the expression of BCL-2 and BCL-XL (Figure 2B). Thus, the reduced growth of tumors in mice xenografted with *TOB1*-transfected MCF-7 cells is partly associated with increased apoptotic cell death [16]. Moreover, treatment of MCF-7 cells with retinoic acid induced the expression of several tumor suppressor genes, including *TOB1*, which may account for the apoptotic induction [44]. Adenoviral-mediated overexpression of TOB1 sensitizes breast cancer cells to radiotherapy and induced apoptosis by preventing DNA repair and inducing the expression of the pro-apoptotic protein BAX (Figure 2B) [45]. Our recent study revealed that TOB1 overexpression in MKN28 and AGS cells shows increased BAX expression, decreased BCL-2 expression, activation of caspase-3, and the cleavage of poly-(ADP ribose) polymerase (PARP), which leads to induction of cell death [29].

In contrast, Suzuki *et al.* [46] suggested that the clearance of TOB1 by proteasomal degradation enhances ultraviolet radiation (UV)-induced keratinocyte apoptosis through the activation of BAX and caspase-3 irrespective of the presence or absence of TP53, suggesting that TOB1 negatively regulates DNA-damage induced apoptosis. The implication of this inhibition of apoptosis by TOB1 lies in the fact that TOB1 prevents unnecessary cell death upon UV-induced damage and allows damaged cells to recover through DNA repair mechanisms. It has been reported that the DNA replication-initiating kinase CDC7 actively inhibits apoptosis in DNA-damaged cells [47,48]. In a recent study, Suzuki *et al.* [49] demonstrated that CDC7 phosphorylates and interacts with TOB1, thereby blocking cullin 4-DNA damage binding protein 1 (CUL4-DDB1)-mediated degradation of TOB1. The stabilization of TOB1 appears to be the cause underlying the CDC7-mediated survival of DNA-damaged cells. This allows the DNA-damaged cells to undergo a repair process. In fact, an elevated level of DNA damage repair proteins such as XRCC1, MRE11, FEN1, and ATM was observed in the TOB1–overexpressing human bronchial epithelial (HBE) cells, which were protected against ionizing radiation- or X-radiation-induced cell death [50]. Thus, it may be concluded that TOB1 selectively induces apoptosis in cancer cells, while it protects normal cells from unwanted cell death.

### 4.3. Anti-Invasive and Anti-Migratory Roles of TOB1

TOB1 decreases the migration, invasion, and metastatic ability of cancer cells [29,51]. Jiao *et al.* [51] reported that TOB1 expression in eight human lung cancer cell lines was decreased and that TOB1 overexpression in these cells attenuated proliferation and metastasis by blocking AKT/PTEN-mediated signaling. In gelatin zymography assay, the activities of matrix metalloproteinase 2 (MMP2) and MMP9 were reduced in *TOB1*-transfected cells. On the other hand, siRNA-mediated knockdown of *TOB1* enhanced MMP activity and cell migration. The expression level of E-cadherin (CDH1), a cell-cell interacting protein as well as α, β, and -γ-catenin is also profoundly affected by variations in TOB1 protein levels. Elevated expression of β-catenin and its target genes, such as genes encoding urokinase plasminogen activator receptor (uPAR) and peroxisome proliferator activator receptor-δ (PPARδ), has also been implicated in the invasion and migration of cancer cells (Figure 2B) [52]. We demonstrated that TOB1 overexpression tends to inhibit the cell migration and invasion on MKN28 and AGS cell lines by decreasing CTNNB protein expression and reporter gene activity and by down-regulating the expression of uPAR and PPARδ [29]. The anti-migratory role of TOB1 is shared by BTG2, which is another member of the PC3/BTG/TOB family proteins. For example, BTG2 overexpression in human lung cancer A549 cells attenuated cell proliferation and invasion through down-regulation of cyclin D1, MMP1, and MMP2 [53]. However, BTG2 has been shown to largely suppress medulloblastoma by promoting the migration of cerebellar precursor cells out of the proliferative neuroepithelium. In this regard, the role of TOB1 in suppressing neuronal cancers is yet to be investigated [54]. As a whole, these findings suggest the common antitumor activity of BTG/TOB family proteins.

## 5. Conclusions

The classic function of tumor suppressor proteins is to negatively regulate the neoplastic transformation of cells. Unfortunately, most of the tumor suppressor proteins experience loss of function due to mutations and epimutations during the course of tumor progression [55]. Hence, the restoration of tumor suppressor gene function is a promising strategy for therapeutic intervention and/or prevention of carcinogenesis. For example, the restoration of a tumor suppressor protein, TP53, is well reported for the discovery of novel cancer chemotherapeutics [56]. Relatively less attention has been given to understanding the mechanisms of function of tumor suppressor proteins as targets for new anticancer therapies as compared to the extensive research regarding those of oncogenes. Altogether, research work from different laboratories using distinct experimental systems undoubtedly proved that TOB1 is a tumor suppressor which negatively regulates the cell cycle and has a significant anti-proliferative activity. Moreover, the role of TOB1 in preventing tumor progression has recently been reported. Considering the tumor suppressor function of TOB1 and the fact that the expression of this protein is reduced during carcinogenesis, it is plausible to consider the development of small molecule inducers of TOB1 as anticancer therapies. However, prior to setting up such an ambitious goal, extensive research is required for the better understanding of TOB1 molecular mechanisms. Based on the recent understanding of TOB1, a putative role of TOB1 may be associated with tumor suppressor SMAD4 and oncogenic CTNNB-mediated signaling, likely including SMAD4-mediated suppression of AURKA-CTNNB activation. In contrast to multiple lines of evidence supporting the tumor suppressor function of TOB1, a recent study uncovered another role of TOB1 in promoting proliferation of estrogen-independent breast cancer cells. Zhang *et al.* reported that TOB1 mediates the survival of estrogen-independent ER-positive breast cancers and its reduction specifically increases G1 arrest and sensitivity to AKT and mTOR inhibitors [57]. While the results of this study are inconsistent with the large pool of experimental findings supporting TOB1’s tumor suppressor role, further studies are necessary to deeply elucidate how TOB1 exerts its different roles on estrogen-dependent *versus* estrogen-independent signaling systems with regard to tumorigenesis. Moreover, in the upcoming years, establishing small molecule inducers of TOB1 activity may be considered as a promising choice for developing new anticancer therapies alongside revealing the biochemistry of TOB1 function as a tumor suppressor.
